# Tirzepatide modulates gut microbiota homeostasis to protect against diabetic kidney disease

**DOI:** 10.3389/fmolb.2025.1715024

**Published:** 2025-12-12

**Authors:** Jun Ma, Mengyuan Tao, Wencheng Zhang, Li Zhou, Henglu Zhang, Fei Li, Hongman Zhang, Di Yao, Weiping Lu, Min Wang

**Affiliations:** 1 Department of Electrophysiology, The Affiliated Huai’an No. 1 People’s Hospital of Nanjing Medical University, Huai’an, Jiangsu, China; 2 Department of Endocrinology and Metabolism, The Affiliated Huai’an No. 1 People’s Hospital of Nanjing Medical University, Huai’an, Jiangsu, China; 3 Department of Endocrinology and Metabolism, Huai’an Industrial Park People’s Hospital, Huai’an, Jiangsu, China

**Keywords:** tirzepatide, GIP/GLP-1 RA, gut microbiota, diabetic kidney disease, glycaemic control

## Abstract

**Purpose:**

This study evaluated the effect of Tirzepatide on metabolic profiles, kidney function, and gut microbiota composition in mice with diabetic kidney disease (DKD) and clarify the relationship between gut microbiota alterations and the renoprotective effects.

**Methods:**

Seven-week-old diabetic db/db mice and db/m controls were randomly assigned to three groups: db/db, db/db-T, and db/m. In the db/db-T group, mice received 10 nmol/kg Tirzepatide injections for a duration of 8 weeks. Biochemical and histopathological analyses were used to assess body weight, blood glucose, lipid profile, hepatic and renal function, and renal histopathological changes in mice. An antibiotic-pretreated group (ABX-db/db-T) was established to explore the impact of gut microbiome depletion on the therapeutic effects of Tirzepatide.The composition of gut microbiota was determined through 16S rRNA gene sequencing to assess microbial differences among groups.

**Results:**

Tirzepatide notably decreased fasting blood glucose (FBG), food intake, body weight, glycated hemoglobin A1c (HbA1c), blood lipid levels, and liver function markers, while improving renal function in mice. The renoprotective effects of Tirzepatide were attenuated following gut microbiota depletion. Microbiota analysis revealed that Tirzepatide could reverse dysbiosis and reshape the gut microbial ecosystem. Tirzepatide treatment raised the proportion of beneficial genera, *Clostridium_sensu_stricto_1* and *Romboutsia,* while reducing potentially pathogenic genera, *Erysipelatoclostridium* and *Bacteroides*. Moreover, these microbiota changes were significantly correlated with serum creatinine and urinary albumin/creatinine ratio.

**Conclusion:**

Tirzepatide improves renal function and metabolic parameters in DKD mice through gut microbiome regulation. The underlying mechanism involves the modulation of gut–renal axis through the optimization of microbial composition, promoting the development of beneficial bacteria while inhibiting harmful microbes. These results establish a foundational understanding for the use of Tirzepatide in DKD and suggest that combined interventions targeting the gut microbiota may have potential clinical value.

## 1 Introduction

Diabetic kidney disease (DKD) is a major microvascular complication of diabetes and the foremost contributor to end-stage renal disease (ESRD), affecting nearly half of type 2 diabetes mellitus (T2DM) patients (Limonte et al., 2022). It can lead to kidney failure and cardiovascular complications, ultimately resulting in premature mortality, and imposing a substantial global disease burden. DKD is characterized by persistent proteinuria alongside a gradual reduction in estimated glomerular filtration rate (eGFR), eventually advancing to ESRD (Bosch et al., 2023). Although the pathophysiological mechanisms of DKD have been partially elucidated, its prevention and treatment remain challenging. This results in persistently high incidence and mortality, as well as a reduced quality of life among patients (Sonmez, 2021). Mechanistically, oxidative stress, inflammatory responses, and activation of the renin–angiotensin system intertwine to promote disease progression (Jin et al., 2023). This further underscores the urgent need to explore effective therapeutic strategies and pharmacological interventions (Francis et al., 2024; Efiong et al., 2025).

Traditional therapy for DKD is mainly aimed at controlling blood glucose, levels blood pressure, and blood lipids, supplemented by anti-inflammatory and anti-fibrotic therapies as well as lifestyle interventions (Zhu et al., 2025; Hiraishi et al., 2024). Recent research indicates that novel pharmacological agents exert beneficial effects on renal metabolism and may confer additional systemic advantages. Tirzepatide is the inaugural dual glucagon-like peptide-1 (GLP-1) and glucose-dependent insulinotropic polypeptide (GIP) receptor agonist. It has been extensively used for managing type 2 diabetes mellitus (T2DM) and obesity (Nauck and D'Alessio, 2022; Hu et al., 2025a). Compared to placebo and GLP-1 receptor agonists, Tirzepatide demonstrates significant advantages in weight reduction and glycemic control. It also improves blood pressure and lipid profiles (Heerspink et al., 2022). Clinical studies have confirmed that Tirzepatide effectively modulates appetite-related indicators, significantly reducing food intake, lowering overall appetite scores, enhancing satiety and satisfaction, and decreasing hunger and food consumption expectations (Heise et al., 2023). In a clinical trial involving a Japanese population, a 15 mg dose of Tirzepatide reduced HbA1c by 33.0 mmol/mol (Inagaki et al., 2022; Kadowaki et al., 2022). Regarding lipid regulation, Tirzepatide has been shown to significantly reduce serum total cholesterol (TC), low-density lipoprotein cholesterol (LDL-C), and triglyceride (TG) levels, while increasing high-density lipoprotein cholesterol (HDL-C) concentrations (Ludvik et al., 2021; Yao et al., 2024; Dahl et al., 2022). Mechanistically, it improves the metabolic microenvironment by modulating lipid metabolism pathways, suppressing oxidative stress, and alleviating inflammatory damage. Based on these multi-target regulatory effects, Tirzepatide exhibits considerable potential in the clinical management of MAFLD and its complications. Tirzepatide also demonstrates potential protective effects in cardiac studies, inhibiting the expression of markers related to high-glucose-induced cardiac fibrosis, hypertrophy, and apoptosis (Taktaz et al., 2024; Liang et al., 2025). In renal studies, long-term Tirzepatide therapy in overweight or obese patients with type 1 diabetes has shown significant improvements in TC, LDL-C, TG, systolic blood pressure, and eGFR (Apperloo et al., 2025). Although preliminary studies have looked into the impact of Tirzepatide (Tian et al., 2025a), its specific protective mechanisms in the kidneys remain to be further explored.

As one of the most essential symbiotic ecosystems in the human body, the gut microbiota plays a pivotal role in energy metabolism, immune regulation, and the maintenance of epithelial barrier homeostasis (Hou et al., 2022; Kamble et al., 2024). Dysregulation of the gut microbiota is recognized as an important driver of various metabolic diseases (Takeuchi et al., 2023). Extensive basic and clinical studies have demonstrated that gut microbiota dysbiosis can influence host metabolic pathways, immune responses, and inflammatory processes, thereby contributing to the onset and progression of diabetes and its complications (Portincasa et al., 2024; Jin et al., 2024). The onset and advancement of DKD are intimately linked to gut microbiota dysbiosis, an essential factor for overall health maintenance (Zhao et al., 2022). Gut microbiota imbalance often involves fewer beneficial bacteria that produce short-chain fatty acids and a rise in harmful species, such as Proteobacteria and Bacteroidetes. These changes exacerbate systemic inflammation, insulin resistance, and renal injury by increasing intestinal permeability and producing pro-inflammatory metabolites like lipopolysaccharides (Li et al., 2023a). Therefore, from a microbial ecological perspective, modulating the gut microbiota has appeared as a possible treatment approach to slow DKD progression (Wang et al., 2022; Hu et al., 2025b). Emerging research indicates that Semaglutide plays a key role in restoring gut microbiome balance by promoting the proliferation of beneficial bacteria, while simultaneously suppressing harmful microbial overgrowth, effectively resetting the gut’s ecological harmony. Additionally, Semaglutide enhances kidney function by regulating gut-derived metabolites to alleviate gut-derived inflammation, and to mitigate diabetes-related cognitive impairment, highlighting its potential therapeutic value in DKD (Sun et al., 2025). Liraglutide enhances Akkermansia levels, influencing gut microbiota in diabetic kidney disease. Tirzepatide markedly alleviates hepatic steatosis in diabetic mice, remodels gut microbiota composition, and alters bile acid metabolism (Zhao et al., 2022). Improvements in relevant metabolic and inflammatory markers suggest that it may indirectly protect metabolism-related organs via the gut microbiota–metabolite pathway (Hu et al., 2025a). However, the specific renal protective effects of Tirzepatide in diabetic patients and the mechanisms underlying its relationship with changes in the gut microbiota are still obscure. Therefore, this research aims to explore the renal protective properties of Tirzepatide in diabetic mice, analyze its effects on the gut microbiome and associated metabolites, and further elucidate whether alterations in the gut microbiota partially mediate these beneficial effects.

## 2 Materials and methods

### 2.1 Animals and grouping design

All mice for the experiment were obtained from Nanjing University’s Model Animal Research Center, including seven-week-old male diabetic C57BL/KSJ db/db mice and littermate control db/m mice. The db/db mice carry a leptin receptor mutation and develop early-onset obesity, insulin resistance, and type 2 diabetes. In contrast, db/m mice are heterozygous for this mutation and do not develop the metabolic phenotype, serving as metabolically normal control animals (Garris, 2004). All mice were housed in the Laboratory Animal Center in regulated environments (21 °C ± 2 °C, 45% ± 10% RH, 12-h light and dark rhythm) with unrestricted access to sterile food and water. Animal studies strictly followed the 3Rs principle. The Huai’an First People’s Hospital’s Animal Ethics Committee granted approval for the experimental protocol. (Approval No.: DW-Y-2024-001-01).

Prior to the experiment, the mice underwent a 1-week acclimatization period. At 8 weeks of age, twelve db/db mice were arbitrarily assigned into two groups: db/db and db/db-T. Six db/m mice served as controls concurrently. The db/db-T mice were administered Tirzepatide intraperitoneally each day (Eli Lilly, Indiana, United States) at 10 nmol/kg over an 8-week period. Both db/m and db/db mice were administered identical saline volumes through intraperitoneal injections during the identical timeframe (Yang et al., 2024a; Ma et al., 2025).

**FIGURE 1:**
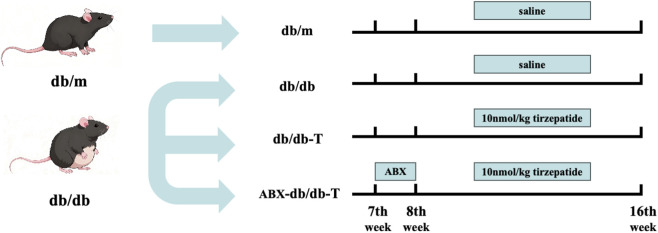
Schematic of the experimental design.

To explore more deeply the impact of the gastrointestinal flora on Tirzepatide’s therapeutic outcomes, an additional ABX-db/db-T group (n = 6) was established by administering a combination of broad-spectrum antibiotics before therapy. One week before the experiment, ABX-db/db-T mice were given daily oral doses of an antibiotic mixture (ampicillin 100 mg/kg, vancomycin 50 mg/kg, neomycin 100 mg/kg, metronidazole 100 mg/kg; Macklin, Shanghai, China) at 0.2 mL for 1 week. Subsequently, these mice were treated with Tirzepatide in the same manner as the db/db-T group (Figure 1).

### 2.2 Biochemical analysis

The mice were weighed weekly using a scale, and all mice were subjected to a 12-h fasting period while having free access to water. Tail vein blood samples were taken, and fasting glucose (FBG) levels were analyzed using a glucometer (OneTouch VerioVue, Johnson and Johnson, United States), Glycated hemoglobin A1c (HbA1c) levels were assessed using a glycated hemoglobin analyzer (BioHermes, Wuxi, China) both before and after the commencement of the study.

Eight weeks after the administration of Tirzepatide, blood samples (1–1.5 mL) were collected via enucleation after the animals were anesthetized. The samples rested at ambient temperature for an hour prior to centrifugation at 3,000 rpm in a refrigerated centrifuge. Serum was separated after 10 min, transferred to fresh sterile tubes and stored at −80 °C. The levels of alanine aminotransferase (ALT), aspartate aminotransferase (AST), TC, TG, HDL-C, LDL-C, blood urea nitrogen (BUN), uric acid (UA) and creatinine (Cr) in the serum of each mouse were measured employing the Roche Cobas c702 automated biochemistry analyzer (Roche Diagnostics, Germany). Simultaneously, the urinary albumin-creatinine ratio (ACR) in the urine of the mice was determined using the same analyzer.

### 2.3 Histopathological analysis

After blood sampling, the mice were humanely euthanized via cervical dislocation. The abdominal cavities were opened, and the kidneys excised and placed in ice-cold PBS buffer solution for rinsing. The capsules were then removed and cleaned before being embedded in 4% paraformaldehyde. The specimens were subjected to gradient dehydration with ethanol, paraffin embedding, and 3 µm microtome sectioning. The sections were dewaxed and washed, then stained with hematoxylin and eosin (HE) and periodic acid–Schiff (PAS). The pathological morphology of renal tissue in each group was examined using light microscopy at 400× magnification.

### 2.4 16S rRNA sequencing

Before the end of the experiment, mice were individually handled under a sterile laminar flow hood to induce defecation by gentle handling. Feces from all three groups were collected into sterile cryovials, with at least 4–5 fecal pellets collected per mouse. A 20 mg fecal sample was weighed and placed into a centrifuge tube. Genomic DNA was extracted using standard procedures. DNA purity and concentration were evaluated through 1% agarose gel electrophoresis, following which the samples were adjusted to a final concentration of 1 μg/μL. The 16S rRNA gene was amplified using barcoded primers. Each PCR reaction contained 15 µL of high-fidelity master mix, 0.2 µM of each primer, and approximately 10 ng of template DNA. PCR products were verified by 2% agarose gel electrophoresis with SYBR Green staining, and the target bands were excised and purified. The products, after purification, were combined in equal molar concentrations and used for library construction. After quality assessment, paired-end sequencing (250 bp) was performed on a high-throughput sequencing platform.

### 2.5 Microbial diversity analysis

Sequences were classified into taxonomic groups using the BLAST tool, referencing the SILVA138 database. Rarefaction curves were generated using QIIME software, version 1.8.0. and indices of richness and diversity were calculated based on ASV data. To compare community membership and structure across samples, heatmaps were constructed for the top amplicon sequence variants (ASVs) using Mothur. Bar plots were created in R (v3.6.0) to visualize taxonomic annotation and relative abundance. Alpha diversity of gut microbiota was evaluated through the Chao1 and Shannon indices method, and beta diversity was characterized via principal coordinate analysis (PCoA) and constrained PCoA (CPCoA). The most abundant species, identified across taxonomic levels, were visualized in a heatmap, and their correlations with metabolic parameters were analyzed.

### 2.6 Statistical analysis

Data analysis was conducted with GraphPad Prism software, version 9.5.1. The quantitative data are presented as mean ± standard deviation (x̄ ± s). To evaluate statistical differences between two or more groups, one-way analysis of variance (ANOVA) was employed. Statistical significance was defined as a P value <0.05.

## 3 Results

### 3.1 Tirzepatide improves metabolic parameters in DKD mice

**FIGURE 2:**
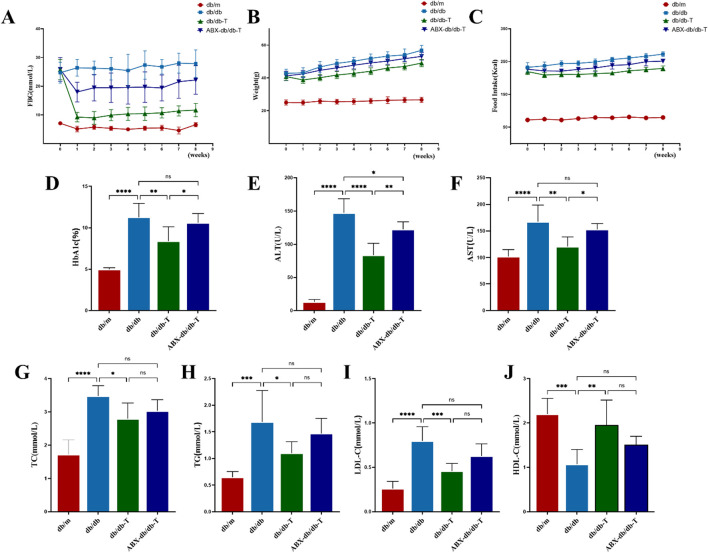
Analysis of metabolic indicators in mice from each group after medication treatment. Each group consisted of 6 mice (n = 6). Values shown as mean ± standard deviation. **(A)** Variations in fasting blood glucose (FBG) among mouse groups. **(B)** Body weight variations across mouse groups. **(C)** Weekly food consumption trends in mice across experimental groups. **(D)** Glycated hemoglobin A1c (HbA1c). **(E)** Alanine aminotransferase (ALT). **(F)** Aspartate aminotransferase (AST). **(G)** Total cholesterol (TC). **(H)** Triglyceride (TG). **(I)** Low-density lipoprotein cholesterol (LDL-C). **(J)** High-density lipoprotein cholesterol (HDL-C). *P < 0.05, **P < 0.01, ***P < 0.001, ****P < 0.0001 and ns: not significant.

During the initial stage of the investigation, db/db mice had considerably higher levels of FBG and HbA1c than db/m controls. By the end of the experiment, these parameters remained elevated in the db/db group, whereas they were markedly reduced in the intervention group, reaching levels markedly below those seen in untreated db/db mice (Figures 2A,D). The weight of db/db mice was much greater than that of db/m controls,and all three groups exhibited an overall increasing trend throughout the study. Following administration of Tirzepatide, the body weight of db/db-T mice decreased dramatically after 1 week of treatment, whereas db/db mice maintained the highest body weight among the three groups (Figure 2B). The db/db mice consumed significantly more food than db/m mice, and during the experiment, food consumption in the db/db group progressively increased, whereas it gradually decreased in the db/db-T group. In contrast, the db/m group showed no noteworthy change in food intake throughout the experimental period (Figure 2C). Further analysis showed that blood lipid and liver function markers (ALT, AST, TC, TG, LDL-C) were markedly higher in db/db mice, whereas HDL-C levels were notably decreased. In contrast, these markers in the db/db-T group revealed significant improvement (Figures 2E–J).

To explore whether the effects of Tirzepatide on metabolic parameters were related to the gut microbiota, db/db mice were pretreated with antibiotics (ABX-db/db-T) to deplete the gut microbiota. The results showed that improvements in food intake, body weight, blood lipids, liver function, FBG, and HbA1c were significantly attenuated in antibiotic-treated db/db mice, suggesting that depletion of gut microbiota weakened the regulatory impacts of Tirzepatide on metabolic function.

### 3.2 Tirzepatide retarded renal progression in DKD mice

**FIGURE 3:**
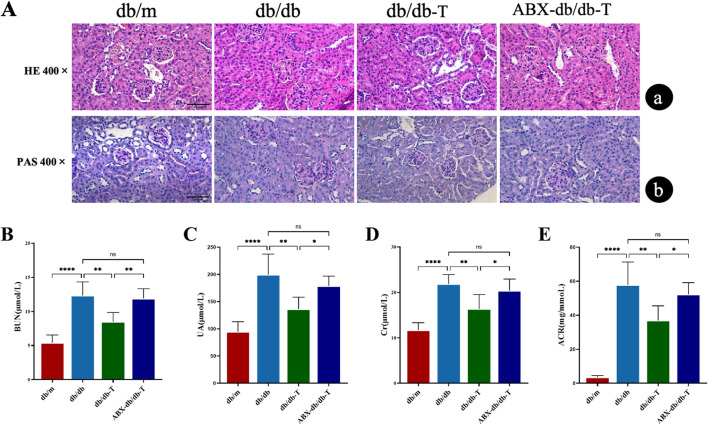
Comparison of renal function markers and histopathology in treated mice across groups. Each group contained 6 mice (n = 6). Results are shown as mean ± SD. **(A)** Representative images of mouse kidneys in each group stained with HE (a) and PAS (b). (original magnification, 400×) **(B)** Blood urea nitrogen (BUN). **(C)** Serum uric acid (UA). **(D)** Serum creatinine (Cr). **(E)** urinary albumin/creatinine ratio (ACR). *P < 0.05, **P < 0.01, ***P < 0.001, ****P < 0.0001 and ns: not significant.

The db/db group had considerably higher levels of BUN, UA, Cr, and ACR at the end of the trial. Histological examination with HE and PAS staining showed glomerular cell proliferation, enlargement of the mesangial matrix, and increase in the thickness of the basement membrane, confirming the proper establishment of the DKD model (Figure 3A). Following Tirzepatide treatment, these indices in the db/db-T group were significantly reduced, indicating a renoprotective effect. Histological assessment demonstrated that Tirzepatide treatment reduced glomerular hypertrophy, attenuated mesangial matrix expansion, decreased basement membrane thickness, and alleviated interstitial fibrosis. However, the renoprotective effects on renal function and histopathology were markedly attenuated in the ABX-db/db-T group versus db/db-T group (Figures 3B–E).

### 3.3 Tirzepatide altered the structure of the gut microbiota in DKD mice

**FIGURE 4:**
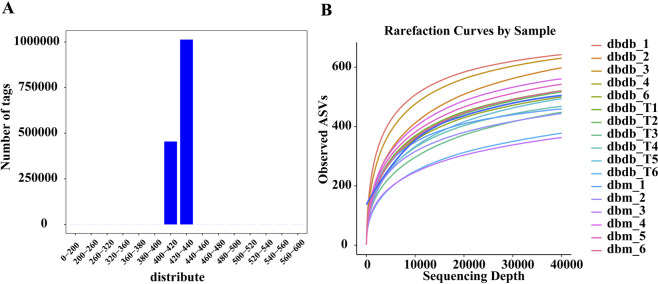
Quality control analysis of the gut microbiota. **(A)** Distribution of the effective sequence length. **(B)** Rarefaction curves.

16S rRNA gene sequencing was used to evaluate the effect of Tirzepatide on the intestinal microenvironment. As seen in Figure 4A, after data preprocessing, the length of high-quality sequences was concentrated at 400–440 bp, meeting experimental requirements. Rarefaction curve analysis indicated that when sequencing depth reached 40,000, the curve plateaued, suggesting that the data had saturated and included all sampled species (Figure 4B).

**FIGURE 5:**
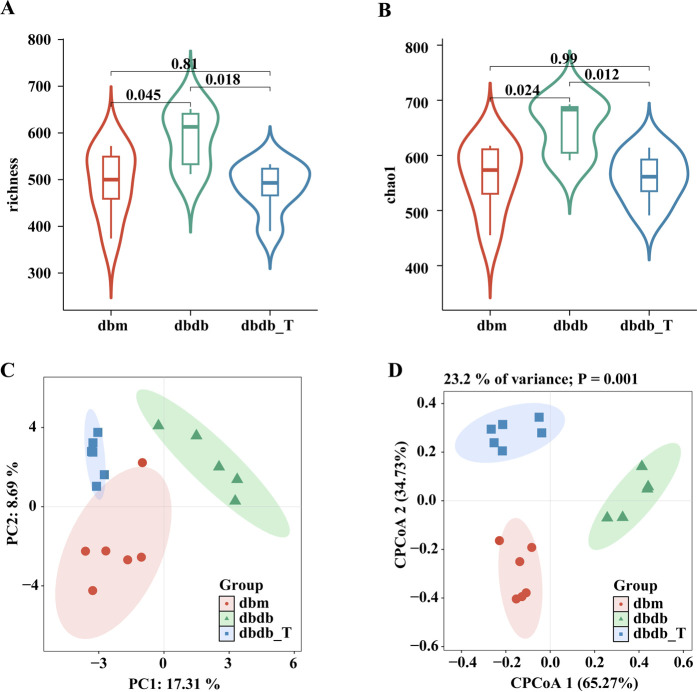
Tirzepatide altered the structure of the gut microbiota. **(A)** Richess index. **(B)** Chao index. **(C)** Principal coordinates analysis (PCoA). **(D)** Constrained principal coordinates analysis (CPCoA).

α-Diversity assessment indicated elevated richness and Chao1 metrics in the db/db cohort versus the db/m cohort, while the db/db-T group exhibited lower diversity, with no discernible variation from the control group, suggesting that Tirzepatide modulated α-diversity and improved microbiota composition (Figures 5A,B). β-Diversity analysis indicated a notable variance in microbial composition across the db/db and db/m cohorts. After treatment, the db/db-T group’s microbial composition became more similar to the db/m group (Figures 5C,D), suggesting that Tirzepatide partially reversed gut microbiota dysbiosis.

### 3.4 Tirzepatide modified the composition of the gut microbiota in DKD mice

**FIGURE 6:**
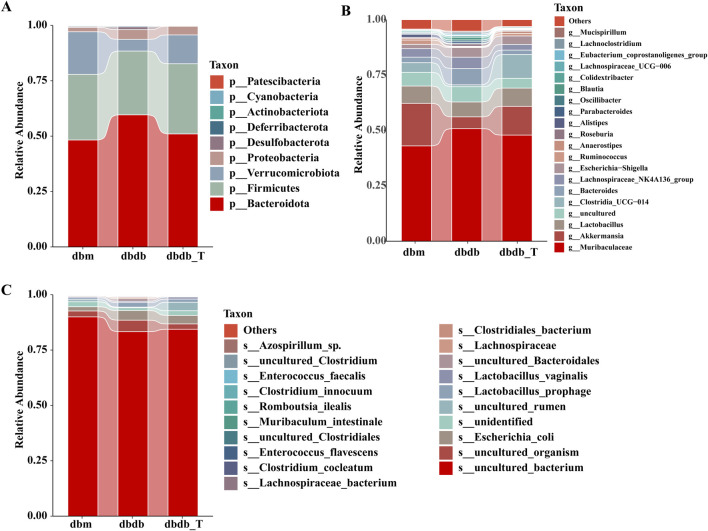
Tirzepatide modified the composition of the gut microbiota. **(A)** Community barplot analysis at the phylum level. **(B)** Community barplot analysis at the genus level. **(C)** Community barplot analysis at the species level.

At the phylum level, *Bacteroidota, Firmicutes,* and *Verrucomicrobiota* were the dominant phyla in all groups: db/m, 48.24%, 29.65%, and 19.20%; db/db, 59.60%, 28.79%, and 5.29%; db/db-T, 51.00%, 31.69%, and 13.00% (Figure 6A). At the species level, *Muribaculaceae, Akkermansia,* and *Lactobacillus* were the dominant genera in the db/m group, accounting for 43.02%, 19.20%, and 7.83%, respectively. In the db/db group, the main genera were *Muribaculaceae, Bacteroides,* and *Lactobacillus,* representing 50.83%, 7.37%, and 6.72%. In the db/db-T group, *Muribaculaceae, Akkermansia,* and *Clostridia_UCG-014* were the predominant genera, with relative abundances of 47.88%, 13.00%, and 10.75% (Figure 6B). At the species level, *Lactobacillus prophage* and *Escherichia coli* were the dominant species across all three groups (Figure 6C).

### 3.5 Genus-level microbiota changes induced by tirzepatide in DKD mice

**FIGURE 7:**
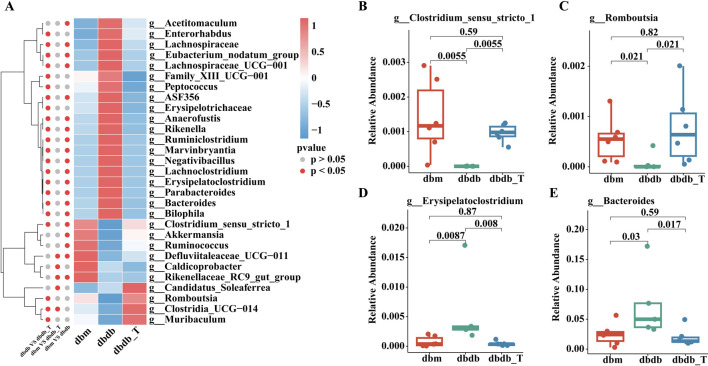
Genus-level analysis of microbiota variations. **(A)** Heatmap illustrating gut microbiota categorized by genus. **(B)** The proportional representation of Clostridium-sensu-stricto-1 within the genus level. **(C)** The proportional presence of *Romboutsia* at the genus level. **(D)** The relative proportion of *Erysipelatoclostridium* within the genus category. **(E)** The relative presence of *Bacteroides* at the genus level.

According to the genus-level community heatmap, the following genera exhibited significant changes after Tirzepatide treatment: *Clostridium_sensu_stricto_1*, *Akkermansia*, *Romboutsia*, Lachnospiraceae, *ASF356, Enterorhabdus*, *Negativibacillus*, *Clostridia_UCG-014*, and *Muribaculum* (Figure 7A; Supplementary Table S1).

*Clostridium_sensu_stricto_1*, *Romboutsia*, and *Akkermansia* abundances were lower in the db/db group than in the db/m group, and these taxa were significantly enriched following Tirzepatide treatment. Conversely, genera such as *Erysipelatoclostridium*, *Bacteroides*, and *Lachnoclostridium* were elevated in the db/db group but notably reduced after treatment, suggesting that Tirzepatide may improve metabolic function by modulating the microbiota (Figures 7B–E).

### 3.6 Correlation between differential intestinal genera and key metabolic indicators in DKD mice

**FIGURE 8:**
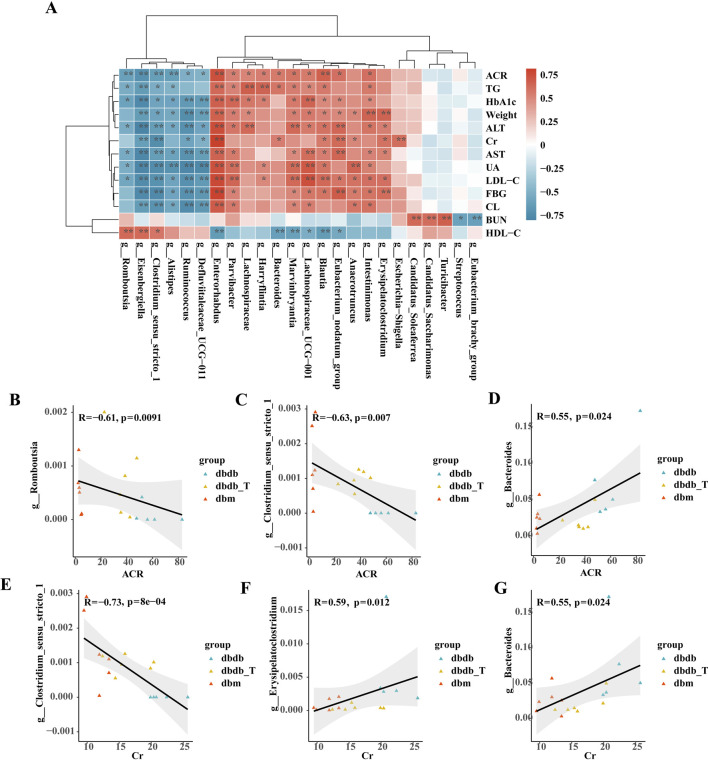
Spearman correlation of gut microbiota and metabolic markers in DKD across groups. **(A)** Heatmap illustrating the correlation analysis between gut microbiota and various parameters is presented. The gradient colors represent the correlation coefficients, with red signifying a positive correlation and blue indicating a negative one. *P < 0.05, **P < 0.01. **(B)** Scatter Plot of *Romboutsia*-ACR Association. **(C)** Scatter Diagram Illustrating the Association between *Clostridium-sensu-stricto-1* and ACR. **(D)** Scatter Diagram Showing the Relationship Between *Bacteroides* and ACR. **(E)** Correlation Scatter Plot between *Clostridium-sensu-stricto-1* and Cr. **(F)** Scatter Plot Illustrating the Correlation Between *Erysipelatoclostridium* and Cr. **(G)** Scatter Diagram Illustrating the Association between *Bacteroides* and Cr.

Correlation analysis indicated that several differential genera were significantly associated with metabolic parameters. *Clostridium_sensu_stricto_1* showed negative correlations with ACR, Cr, and multiple metabolic markers (ALT, AST, UA, LDL-C, FBG); *Romboutsia* was negatively correlated with ACR and positively with HDL-C; *Erysipelatoclostridium* was positively correlated with body weight, Cr, ALT, AST, LDL-C, and FBG; *Bacteroides* showed favorable associations with ACR, Cr, TG, and a negative correlation with HDL-C. Multiple genera were significantly correlated with Cr and ACR, suggesting their potential role in regulating renal function and metabolic status (Figure 8).

### 3.7 The effect of tirzepatide treatment on metabolic pathways

**FIGURE 9:**
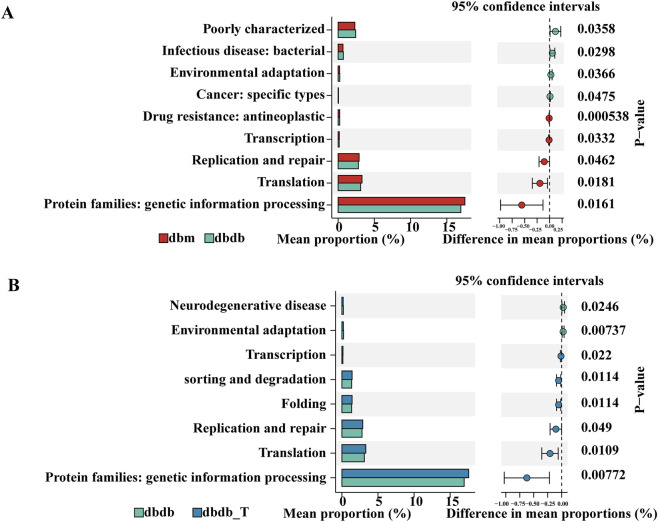
KEGG pathway analysis of different gut microbiota. **(A)** Comparison between the db/m and db/db groups. **(B)** Comparison between the db/db and db/db-T groups.

KEGG pathway analysis revealed functional differences in microbiomes between groups. Eight pathways differed significantly between the db/db and db/m groups: the db/db group was enriched in pathways related to “infectious disease: bacterial” and “environmental adaptation,” whereas the db/m group was enriched in pathways associated with “transcription,” “replication and repair,” and “translation,” which are involved in genetic information processing. After Tirzepatide treatment, the db/db-T group showed enrichment in pathways related to “sorting and degradation,” “folding,” and multiple genetic information–processing functions suggesting that Tirzepatide may improve microbial function by modulating these key pathways (Figure 9).

## 4 Discussion

DKD is the most prevalent and severe chronic complication of diabetes and a primary contributor to end-stage renal disease, thereby posing a substantial burden on global public health (Tian et al., 2023). Although interventions such as glycemic and blood pressure control, renin–angiotensin–aldosterone system (RAAS) inhibitors, and sodium–glucose cotransporter 2 (SGLT2) inhibitors can partially slow disease progression, these therapeutic strategies remain limited, particularly in patients with impaired renal function or significant proteinuria, where reversing pathological alterations or fully restoring glomerular and tubular structure and function is challenging (Yu et al., 2024). In recent years, accumulating evidence has demonstrated a strong connection between the gut microbiota and metabolic diseases, including diabetes and its complications, with the gut–kidney axis emerging as a potentially novel therapeutic target (Mao et al., 2023).

This study demonstrated that Tirzepatide reduced blood glucose and body weight and improved hepatic and renal function, consistent with previous reports (Sattar et al., 2022; Frías et al., 2021). Meanwhile, Tirzepatide reversed gut microbiota dysbiosis, and shifts in specific taxa were significantly correlated with metabolic parameters. Notably, depletion of the gut microbiota markedly attenuated the metabolic benefits of Tirzepatide in db/db mice, suggesting that its modulation of the gut microbiota may constitute one of the key mechanisms underlying its renoprotective effects. Previous studies have shown that Tirzepatide can improve insulin sensitivity and maintain glucose homeostasis by activating GLP-1 and GIP receptors, thereby indirectly alleviating diabetes-induced hyperglycemia and subsequent renal injury (Samms et al., 2021). Additionally, Tirzepatide alleviates renal inflammation and oxidative stress (Yang et al., 2025), inhibits apoptosis of glomerular and tubular cells, and reduces urinary protein excretion and serum creatinine levels (Tian et al., 2025a). Furthermore, Tirzepatide regulates lipid metabolism, thereby mitigating lipotoxicity-associated renal injury (Caruso and Giorgino, 2024). Tirzepatide enriches beneficial microbial species while suppressing potentially pro-inflammatory microbes. This optimizes the gut microbial ecosystem. It may also modulate host inflammation and oxidative stress through gut-derived metabolites, contributing to a gut–kidney axis protective effect (Wang et al., 2025). In summary, Tirzepatide may confer renoprotective effects in DKD mice through multiple synergistic mechanisms. These include improving glucose and lipid metabolism, attenuating inflammation and oxidative stress, and modulating the gut microbiota.

This study demonstrated that Tirzepatide partially restored gut microbiota balance in mice with diabetic kidney disease, improving microbial homeostasis and ameliorating the progression of diabetic renal pathology. After Tirzepatide treatment, the abundances of *Akkermansia, Clostridium_sensu_stricto_1*, and *Romboutsia* increased significantly. In contrast, the abundances of *Erysipelatoclostridium* and *Bacteroides* decreased markedly. These differentially abundant genera were significantly correlated with various metabolic parameters, suggesting that they may exert important effects on host health by modulating renal function and metabolic status. Specifically, *Akkermansia* has been shown to alleviate low-grade chronic intestinal inflammation and improve gut barrier function (Mo et al., 2024). By modulating lipid metabolism, *Akkermansia* can reduce the development of obesity and metabolic syndrome, and is thus considered a candidate for the “next-generation probiotic” (Niu et al., 2024; Pellegrino et al., 2023). The genus *Romboutsia* has been positively associated with skeletal muscle antioxidant capacity and glucose metabolism (Zhang et al., 2023). Previous studies have reported that supplementation with *Romboutsia s*trains can restore gut barrier function in type 2 diabetic mice by producing short-chain fatty acids and reducing levels of pro-inflammatory cytokines and lipopolysaccharides (Ye et al., 2024). Specific species within the *Clostridium_sensu_stricto_1* group are capable of producing short-chain fatty acids, such as butyrate, which are often reduced during metabolic disturbances including fat accumulation and liver fibrosis (Yin et al., 2023; Lanthier and Delzenne, 2022). Supplementation with synbiotics can promote the enrichment of the probiotic *Clostridium_sensu_stricto_1* and activate functional pathways related to amino acid and short-chain fatty acid biosynthesis (Li et al., 2023b). Whole-genome sequencing database analyses have shown that *Clostridium_sensu_stricto_1 e*xerts a protective effect against the progression of diabetic kidney disease (Lin and Chen, 2024). In contrast, this study found that the abundances of *Erysipelatoclostridium* and *Bacteroides* were significantly increased in samples with metabolic disturbances. Previous studies have frequently associated these taxa with adverse metabolic phenotypes or inflammatory states. Obesity-related indices and BMI are positively correlated with *Erysipelatoclostridium* (Zhu and Hou, 2024; Yang et al., 2024b; Chen et al., 2023), and this genus is considered a pathogenic contributor to obesity (Sun et al., 2018). Prospective cohort studies have shown that *Erysipelatoclostridium* is negatively correlated with fasting glucose, insulin levels, and insulin resistance. The proliferation of *Erysipelatoclostridium,* particularly *E. ramosum,* may exacerbate high-fat diet–induced obesity and metabolic disturbances by disrupting the gut barrier and activating inflammatory pathways such as TLR4 (Chen et al., 2023). Multiple microbiome studies in CKD and end-stage renal disease (ESRD) have reported increased abundances of *Erysipelatoclostridium* in patients with impaired renal function or undergoing dialysis, suggesting a potential association with uremic metabolites, inflammation, or the gut–kidney axis (Qin et al., 2025). *Bacteroides,* a common anaerobic genus in the gut, produces metabolites closely linked to host metabolic disturbances. Studies have shown that its abundance is positively correlated with insulin resistance (Zeng et al., 2019) and may exacerbate the progression of metabolic-associated fatty liver disease (MAFLD) by modulating lipid metabolism (Huang et al., 2024). Other studies have reported a significant enrichment of *Bacteroides stercoris i*n fecal samples from patients with diabetic kidney disease, with notable associations with hepatic triglyceride levels and liver enzyme indicators (Ni et al., 2023). In summary, the observed shifts in beneficial and detrimental bacterial taxa suggest that these genera may serve as potential targets for alternative or adjunctive interventions in metabolic disorders.

Future studies need to further explore the mechanisms by which Tirzepatide improves DKD through the gut microbiome. Tirzepatide significantly increased the abundance of *Clostridium_sensu_stricto_1* and *Romboutsia.* These taxa were closely associated with renal function markers, suggesting that they may regulate the gut–kidney axis. Therefore, future studies could utilize antibiotic pre-treatment and fecal microbiota transplantation experiments to validate the causal relationship between microbiome changes and renal effects. By depleting the gut microbiota in db/db mice and transplanting feces from the Tirzepatide-treated group and the control group, changes in renal function post-transplantation will be observed to confirm whether ecological changes in the microbiome directly affect renal function (Zhong et al., 2023). Additionally, single-strain or defined microbiota colonization experiments will help validate the renal protective effects of specific microbial communities (Zhou et al., 2022). By colonizing *Clostridium_sensu_stricto_1* and *Romboutsia*, we aim to investigate whether these microbes can improve metabolic and renal function in the absence of Tirzepatide intervention. Additionally, the study will explore whether the combined use of Tirzepatide enhances its renal protective effects (Yasuma et al., 2025). To further explore the mechanisms by which the gut microbiome regulates the host through metabolites, future studies will integrate metagenomics and metabolomics (e.g., short-chain fatty acids, bile acid analysis) to assess the functional changes in the gut microbiota following Tirzepatide treatment (Tian et al., 2025b; Kamble et al., 2025). Accumulating evidence indicates that short-chain fatty acids can directly suppress renal inflammation and fibrosis (Udomkarnjananun et al., 2025). Therefore, metabolite supplementation or receptor antagonism experiments could be employed to further determine whether these metabolites mediate the renoprotective effects induced by Tirzepatide (Corte-Iglesias et al., 2024). Finally, *in vitro* experiments will validate the above mechanisms at the cellular level. Intestinal epithelial and renal tubular cell models are commonly used to investigate the barrier-protective and anti-inflammatory effects of microbial metabolites (Yao et al., 2025). These models could therefore be applied to test the direct effects of culture supernatants or metabolites derived from *Clostridium_sensu_stricto_1* and *Romboutsia* on host cells. Particular attention will be given to whether these treatments improve intestinal barrier function and inhibit the expression of inflammation and fibrosis markers. Through these experiments, the microbiome-mediated mechanisms underlying the renal protective effects of Tirzepatide are expected to be elucidated. These findings would also provide theoretical support for future microbiome-based interventions used in combination with Tirzepatide.

This study has several limitations. First, Although antibiotic treatment helped assess microbiota-dependent effects, it may also induce metabolic changes. In addition, tirzepatide-induced changes in body weight and blood glucose, although primary outcomes, may still partially influence renal outcomes. The limited set of control groups also restricts the interpretation of underlying mechanisms. Future studies should address these limitations to provide a clearer causal understanding. Second, this study was primarily conducted in mouse models, and whether these findings can be fully extrapolated to human patients requires further validation. Third, the safety, potential resistance, and side effects associated with long-term use of Tirzepatide require further validation in clinical trials.

This study’s originality comes from its first demonstration that Tirzepatide may exert additional renal protective effects in DKD models by modulating the gut microbiota. This discovery not only advances existing research on how Tirzepatide acts in diabetes and its related complications, but also provides novel theoretical underpinnings for future interventions combining targeted gut microbiota manipulation with pharmacological treatments.

## Data Availability

The datasets presented in this study can be found in online repositories. The names of the repository/repositories and accession number(s) can be found below: https://www.ncbi.nlm.nih.gov/, PRJNA1335138.
