# Supplementary material for: Tirzepatide modulates gut microbiota homeostasis to protect against diabetic kidney disease
Source: Front Mol Biosci. 2025 Dec 12;12:1715024. doi: 10.3389/fmolb.2025.1715024 (PMC12740902; doi:10.3389/fmolb.2025.1715024)
Supplement: Supplementary file 1 [file Table1.pdf]

Table S1. Relative Abundance of Key Gut Microbial Taxa Across Experimental Groups

Taxon	**db/m (mean ± SE, %)**	**db/db (mean ± SE, %)**	**db/db_T (mean ± SE, %)**
p__Bacteroidota	48.239±3.342	59.602±3.635	50.984±4.566
p__Firmicutes	29.648±3.096	28.788±5.369	31.692±1.844
p__Verrucomicrobiota	19.2±3.392	5.293±2.811	13±4.083
g__ASF356	0.014±0.005	0.045±0.015	0.003±0.001
g__Akkermansia	19.2±3.392	5.293±2.811	13±4.083
g__Bacteroides	2.468±0.759	7.365±2.576	1.983±0.613
g__Candidatus_Soleaferrea	0.009±0.002	0.011±0.002	0.019±0.004
g__Clostridia_UCG.014	4.359±1.66	0.834±0.508	10.752±1.848
g__Clostridium_sensu_stricto_1	0.142±0.045	0±0	0.097±0.01
g__Enterorhabdus	0.052±0.014	0.154±0.012	0.087±0.011
g__Erysipelatoclostridium	0.078±0.036	0.561±0.287	0.042±0.016
g__Lachnoclostridium	0.095±0.028	0.859±0.322	0.037±0.011
g__Lachnospiraceae	0.001±0.001	0.009±0.003	0.003±0.001
g__Lactobacillus	7.825±2.593	6.717±2.491	8.228±1.812
g__Muribaculaceae	43.015±3.043	50.832±3.38	47.879±4.43
g__Muribaculum	0.207±0.059	0.073±0.019	0.374±0.116
g__Negativibacillus	0.01±0.004	0.059±0.021	0.007±0.003
g__Romboutsia	0.055±0.018	0.009±0.008	0.077±0.03
s__Escherichia_coli	1.913±1.47	4.358±1.464	3.838±0.953
s__Lactobacillus_prophage	0.826±0.246	2.056±1.597	1.414±0.535
